# A review of the presentation and outcome of sarcoidosis in coronavirus disease 2019

**Published:** 2021-09-27

**Authors:** Lina James George, Anil Mathew Philip, Kevin John John, Anu Anna George, Jemimah Nayar, Ajay Kumar Mishra, Amos Lal

**Affiliations:** ^1^Department of Pulmonary Medicine, DR KM Cherian Institute of Medical Sciences, Kallissery, Kerala, India; ^2^Department of Medicine, St. Thomas Mission Hospital, Kattanam, Kerala, India; ^3^Department of Critical Care, Believers Church Medical College Hospital, Thiruvalla, Kerala, India; ^4^Department of Internal Medicine, Saint Vincent Hospital, Worcester, Massachusetts, United States; ^5^Department of Nuclear Medicine, Christian Medical College, Vellore, Tamil Nadu, India; ^6^Department of Internal Medicine, Division of Cardiology, Saint Vincent Hospital, Worcester, Massachusetts, United States; ^7^Department of Medicine, Division of Pulmonary and Critical Care Medicine, Mayo Clinic, Rochester, Minnesota, United States

**Keywords:** coronavirus disease 2019, sarcoidosis

## Abstract

**Background:**

In the setting of the current pandemic, concerns have arisen regarding the multisystemic involvement of sarcoidosis and the possible exacerbations in response to the exposure to severe acute respiratory syndrome coronavirus 2.

**Aim:**

This study aims to compare the differences in clinical presentation, management, and outcome of coronavirus disease 2019 (COVID-19) between patients with sarcoidosis and those in the general population.

**Methods:**

A literature search was conducted by reviewing original research articles such as case reports, case series, observational studies, and questionnaire-based surveys published in PubMed/Medline, Web of Science, and Google scholar. Data from individual patients in case series and case reports have been pooled to create a data set that was compared with larger such cohorts obtained from several other observational studies.

**Results:**

Twenty-seven patients were identified from 14 original articles. No significant differences were found in the clinical manifestations of patients with sarcoidosis presenting with COVID-19 as compared to the general population. The rate of hospitalization in our study was found to be 48.1%. The overall mortality in our study was 7.4%, which is higher than the global average of 2.1%.

**Conclusion:**

Our observations have reinforced the hypothesis that the presence of additional medical comorbidities is associated with a higher risk of intensive care unit admission. Furthermore, the presence of moderate to a severe limitation in pulmonary functions is an additional risk factor associated with increased hospital admissions and mortality in sarcoidosis. However, neither the diagnosis of sarcoidosis nor ongoing treatment with steroids, methotrexate, or other immunosuppressants was associated with a poorer prognosis in patients with sarcoidosis.

**Relevance for patients:**

Patients with sarcoidosis must take added precautions to mitigate the risk of acquiring COVID-19 infection in view of the COVID-19-related mortality rate in this group of patients. Specifically, immunocompromised patients (on immunomodulator drugs and high dose steroids) have been found to have an increased risk of contracting COVID-19. Overall impact on prognostication and outcome in cases requiring hospitalization remains yet to be determined.

## 1. Introduction

Coronavirus disease 2019 (COVID-19) was first identified in Asia in December of 2019 and continues to be a public health emergency of international concern. As of the 16^th^ of August 2021, the severe acute respiratory syndrome coronavirus 2 (SARS-CoV-2) has infected more than 207 million individuals worldwide and has caused over 4.31 million deaths. As with most viral illnesses, the incubation period of COVID-19 is approximately 14 days, but most symptomatic infections occur around 4-5 days after exposure [1,2]. In different virus variants, the most common symptoms in the order of occurrence are cough, fever, myalgia, headache, dyspnea, sore throat, diarrhea, nausea or vomiting, loss of smell, abdominal pain, and rhinorrhea [3]. Although the disease may be asymptomatic in some, older age and preexisting medical comorbidities contribute to increased hospital admissions and mortality among those affected by COVID-19. The prevalence of chronic respiratory illness among patients with COVID-19 has been found to range between 1 and 33% [4]. Sarcoidosis is a chronic granulomatous disease that has a relapsing and remitting course often necessitating the induction of glucocorticoid or immunosuppressant-based treatment regimes [5]. The prevalence of lung involvement in sarcoidosis is >90% [6]. The overall rate of COVID-19 infection among sarcoidosis patients recorded on a survey questionnaire ranged from 0.8 to 4.76% [7]. Although it has been hypothesized that the lack of a hyperimmune response to the SARS-CoV-2 antigen may be protective in patients receiving immunomodulators, clinicians cannot be complacent as the effects of immune suppression have not fully been studied [8]. As we continue to reel under the pandemic, varied and protean manifestations of either disease continue to raise questions regarding the most appropriate management both in the acute care setting and in long-term follow-up.

## 2. Methods

### 2.1. Eligibility criteria

In this study, we have included all patients with a pre-existing diagnosis of sarcoidosis who have presented with COVID-19. Case reports and case series with individual patient details have been pooled together for an assessment of clinical manifestations, radiological presentation, and outcomes. Patients with new-onset granulomas mimicking sarcoidosis post-COVID-19 have not been included in the data analysis.

### 2.2. Selection strategy

**Figure 1:**
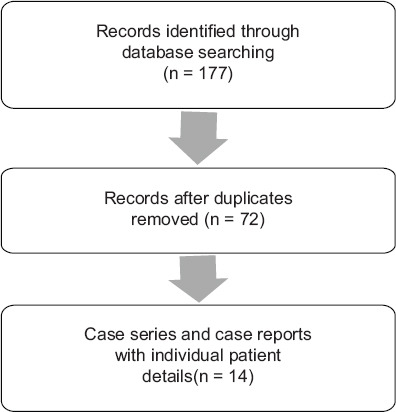
PRISMA flow chart of search

In this review, we included articles on COVID-19 and sarcoidosis published in PubMed/Medline, web of science, and Google scholar till August 14, 2021. We used the search terms “COVID-19” and “Sarcoid” or “2019 nCoV” and “sarcoid” or “COVID 19” and “sarcoidosis” or “2019-nCoV” and “sarcoidosis” or “SARS-CoV-2” and “Sarcoid,” or “SARS-CoV-2” and “Sarcoidosis” in the MeSH database. In Google Scholar, the terms “COVID-19 and sarcoidosis” were used with articles sorted by date. The search terms “Sarcoidosis and COVID-19” were used in Web of science research assistant. A total of 171 articles were identified (Figure 1). After eliminating duplicated articles and non-English articles, a total of 14 articles (both case series and case reports) were identified that met the inclusion criteria all case reports and case series were pooled and analyzed. The findings of this analysis were compared to the other studies reported in the literature. Two independent clinicians were involved in the screening of the articles.

## 3. Results

Fourteen articles described a total of 27 patients presenting with COVID-19 along with sarcoidosis (Table 1). The results of the pooled analysis of case reports and case series are presented below. The mean age of the patients was 53.3 years and 40.7% of them were male. A majority of the patients were from Iran and the USA.

**Table 1:** Summary of cases of patients with sarcoidosis and COVID-19

Sl. No.	Reference	Age	Sex	Details of sarcoidosis and other comorbidities	Chest imaging	Management and outcome
1	Gemcioglu *et al*. [9]	65	F	No details of sarcoidosis mentioned; COPD, HTN, HFpEF	CXR: Chronic fibrosis due to chronic pulmonary disease and peripherally located ground-glass opacities in lungs bilaterally	Required ICU admission. Final outcome-not mentioned
2	Ng *et al*. [10]	78	F	Sarcoidosis present, no details of treatment; hypertension, CVA, type 2 diabetes mellitus.	CXR: Diffuse bilateral patchy opacities, without cardiomegaly suggestive of ARDS	Intubated for impending respiratory failure. Withdrawal of care done
3	Györfi *et al*. [11]	50	M	Lofgren syndrome, treated with steroids (1 year)	CT: Bilateral, peripheral, ill-defined ground-glass opacities involving mainly the right lower lobe, consolidations in right peripheral lung base and the left lower lobe.	Uneventful hospital course. Discharged at 5 days
4	Padala *et al*. [12]	57	F	Stage I pulmonary sarcoidosis, cardiac sarcoidosis with nonischemic cardiomyopathy; complete heart block, and ventricular tachycardia status post biventricular pacemaker with implantable cardioverter-defibrillator, EF 37%. Had been on adalimumab, Methotrexate, prednisolone, amiodarone and mexiletine	CXR: Mild pulmonary vascular congestion but was negative for consolidations or infiltrates	Required ICU admission, mechanical ventilation. Pt improved and was discharged without any oxygen requirement
5	Yates *et al*. [13]	71	F	9-year history of pulmonary sarcoidosis treated with intermittent steroids	CXR: Evidence of chronic lower lung changes attributed to sarcoidosis	Self-monitored pulse oximetry. No oxygen requirement mentioned
6	Bénézit *et al*. [14]	40	M	Pulmonary sarcoidosis was diagnosed 5 yrs back, on HCQ	CT: Diffuse ground-glass opacities, superimposed on the baseline sarcoidosis lesions	Hospitalised.No oxygen requirement mentioned
7	Manansala *et al*. [15]	45	M	Pulmonary sarcoidosis, on Methylprednisolone 8 mg daily; bronchial asthma	CXR: No acute cardiopulmonary process	No hospital admission required
8	Manansala *et al*. [15]	62	F	Advanced pulmonary sarcoidosis, on methotrexate, HCQ, and methylprednisolone; Pulmonary hypertension	CXR: Diffuse advanced interstitial lung disease	Did not require hospitalization, complete recovery at 2-week follow-up
9	Manansala *et al*. [15]	50	M	Ocular, cardiac sarcoid; Uncontrolled hypertension, uncontrolled diabetes, smoker	CXR: Bibasilar reticular infiltrates, later progressed to bilateral pulmonary opacities consistent with ARDS	Required ICU admission. Death due to pulmonary embolism
10	Manansala *et al*. [15]	48	F	Neurologic sarcoid; Uncontrolled hypertension	CXR: Bibasilar atelectasis, bilateral pulmonary opacities	Required ICU admission. Improved and discharged
11	Manansala *et al*. .[15]	46	M	Testicular sarcoid, on methotrexate and infliximab; Uncontrolled hypertension, smoker	CXR: No acute cardiopulmonary process	No hospital admission required
12	Kiani *et al*. [16]	31	M	Prednisolone, Methotrexate	-	No hospital admission required
13	Kiani *et al*. [16]	46	F	Prednisolone	-	No hospital admission required
14	Kiani *et al*. [16]	50	F	Prednisolone, methotrexate, HCQ, diabetes	-	No hospital admission required
15	Kiani *et al*. [16]	53	F	Prednisolone	-	No hospital admission required
16	Kiani *et al*. [16]	43	M	Prednisolone, Methotrexate, ischemic heart disease	-	No hospital admission required
17	Kiani *et al*. [16]	34	F	Prednisolone, methotrexate, HCQ	-	No hospital admission required
18	Kiani *et al*. [16]	53	F	Prednisolone; HTN	-	No hospital admission required
19	Kiani *et al*. [16]	51	F	Prednisolone; hypothyroid	-	No hospital admission required
20	Kiani *et al*. [16]	48	M	Prednisolone, HCQ	-	No hospital admission required
21	Kiani *et al*. [16]	42	M	Prednisolone,	-	No hospital admission required
22	Opoka *et al*. [17]	69	M	Stage II sarcoidosis, not receiving any treatment	CXR: New, bilateral consolidations in the middle and lower lung zones. CT: Diffuse ground-glass opacities localized in middle and lower parts of the lungs on superimposed ILD and lymphadenopathy.	Required supplemental oxygen. Improved and discharged
23	Ramdani *et al*. [18]	66	F	Pulmonary sarcoidosis of 4 yrs, depressive disorder on amitriptyline. Not on regular hospital follow-up	CT: Hilar and mediastinal Lymphadenopathy, reticular opacities, traction bronchiectasis, ground-glass opacities	No oxygen requirement. Improved and discharged on day 5
24	Chopra *et al*. [19]	42	M	Sarcoidosis, HTN	CT-angio- saddle pulmonary embolus, Venous Doppler- superficial femoral thrombosis	Was initially hemodynamically unstable. Discharged on day 5
25	Bajaj *et al*. [20]	58	F	Stage 4 sarcoidosis on daily prednisolone, DM	CT- B/L GGO with consolidation, CORADS -6	Was admitted to the ICU; Improved and discharged on day 22
26	Kiana *et al*. [16]	88	F	HTN, CAD, sarcoidosis, Polymyalgia rheumatica on HCQ	no data	Hospitalized for 1 day. No oxygen requirement
27	Van Dijck *et al*. [21]	54	F	Sarcoidosis on daily prednisolone and weekly methotrexate	No pulmonary embolism on CTPA	Required hospitalization and supplemental oxygen. Improved and discharged by day 10

F: Female; M: Male; CXR: Chest X-ray; CT: Computed tomography; COPD: Chronic obstructive pulmonary disease; HTN: Hypertension; HFpEF: Heart failure with preserved ejection fraction; ICU: Intensive care unit; HCQ: Hydroxychloroquine; CVA: Cerebrovascular accident; ARDS: Acute respiratory distress syndrome; MODS: Multiorgan dysfunction syndrome; ILD: Interstitial lung disease

### 3.1. Clinical features

All the 27 patients identified in these case reports and case series were diagnosed to have sarcoidosis. Among these patients, nine patients had evidence of pulmonary involvement of sarcoidosis, most often described as fibrosis, with or without accompanying lymphadenopathy on chest imaging. Staging of the disease was provided only in three of the patients. Five patients had extrapulmonary involvement in the form of cardiac, testicular, ocular, neurological, or articular involvement. The average duration of the disease, which was reported in 15 of these patients, was 5 years. The most common symptoms noted among patients in our review were fever (40.7%), myalgia (40.7%) cough (37%), dyspnea (37%), and decreased appetite (18.5%). Atypical symptoms such as diarrhea were seen in four patients (14.8%), ankle swelling in one patient with preexisting Lofgren’s syndrome (3.7%), and anosmia in two patients (7.4%). One patient did not have any symptoms.

### 3.2. Investigations

In 48.14% of patients, the diagnosis of COVID was made with reverse transcription-polymerase chain reaction (RT-PCR). One case series did not report the modality of diagnosis. The most common modality of chest imaging was the chest roentgenogram, which was performed in 37% of the patients. Computed tomography (CT) scan of the chest was performed in six (22.2%) patients. Among them, bilateral ground-glass opacities were noted in nine (33.3%) patients, all of whom were hospitalized. One of the patients with cardiac sarcoidosis-associated-cardiomyopathy had evidence of pulmonary edema. Among the patients who died, findings suggestive of acute respiratory distress syndrome (ARDS) were noted on the chest roentgenogram.

### 3.3. Management and outcome

Of the 27 patients, six (22.2%) were admitted to the intensive care unit (ICU), all of whom had medical comorbidities such as diabetes mellitus, systemic hypertension, old cerebrovascular accident, and chronic obstructive pulmonary disease. Two of these patients (7.4%) died-one due to pulmonary embolism and another due to withdrawal of support on compassionate grounds (Table 2). A noteworthy mention is that three of the six patients who required ICU admission (50%) had extrapulmonary sarcoidosis in the form of cardiac, ocular, and neurological involvement.

**Table 2:** Comparison of studies reporting outcomes of COVID-19 in patients with sarcoidosis

Variables	Our review *n* (%)	Jeny *et al*. [22] *n* (%)	Baughman *et al*. [7] *n* (%)	Morgenthau *et al*. [23] *n* (%)	Hadi *et al*. [24] *n* (%)	Brito-Zerón *et al*. [25] *n* (%)	Baughman *et al*. [26] *n* (%)	Desbois *et al*. [27] *n* (%)
Number of patients	27	36	116	37	954	45	77	26
Patients on steroids	17 (62.9)	25 (69)	36 (31)	6 (16.2)	544 (57)	15	32 (41.6)	84.6
Patients on HCQ	5 (21.7)	3 (8)	8 (7)	Not known	Not known	Not known	9 (11.7)	11.5
Patients on cytotoxic medication	8 (29.6)	14 (39)	27 (23)	7 (18.9)	57 (6)	7	24 (31.2)	38.4
Patients on TNFα inhibitors	2 (7.4)	6 (17)	8 (7)	1 (2.7)	Not known	0	15 (19.5)	3.8
Hospitalizations	13 (48.1)	28 (78)	18 (15.8)	22 (59.5)	185 (19.4)	14 (31.1)	19 (24.7)	4 (26%)
ICU admissions	6 (22.2)	13 (36)	4 (3.4)	Not known	66 (6.9)	2 (4)	6 (7.8)	2 (7.6)
Deaths	2 (7.4)	5 (14)	Not known	6 (16.2)	41 (4.3)	4 (8)	1 (1.3)	2 (7.6)

HCQ: Hydroxychloroquine; TNFα: Tumor necrosis factor α; ICU: Intensive care unit

Among the 27 patients, 11 (40.7%) were only on glucocorticoids for their long-term management. Eight patients were on methotrexate and two of them received additional tumor necrosis factor alpha (TNFa) inhibitors such as infliximab and adalimumab. One of the patients who received adalimumab and one patient on daily prednisolone required ICU admission. However, details of ongoing treatment were not reported for four of the six patients who required ICU admission.

## 4. Discussion

The present coronavirus pandemic has stressed healthcare systems worldwide. As we struggle to come to terms with the scale of the pandemic, the collateral damage in terms of disruption of care of chronic diseases is becoming more evident. Nowhere is this more relevant than in the case of chronic lung diseases like sarcoidosis. The interaction between COVID-19 and sarcoidosis is an interesting one. Many of the patients with sarcoidosis are on long-term immunosuppressive therapy. Although it has been hypothesized that the lack of a hyperimmune response to the SARS-CoV-2 antigen may be protective in patients receiving immunomodulators, hard evidence is lacking. Furthermore, chronic lung damage and reduced pulmonary reserve in patients with pulmonary sarcoidosis may lead to worse outcomes in SARS CoV-2 infection. In this narrative review, we sought to review and consolidate the current literature on this subject.

In our review, it was observed that the clinical presentation, imaging studies, and outcomes of COVID-19 in sarcoidosis did not vary considerably from non-sarcoidosis patients. The most common symptoms noted in the patients were fever (40.7%), myalgia (40.7%) cough (37%), dyspnea (37%), and decreased appetite (18.5%), which is similar to those noted in the general population. Symptoms of COVID-19, such as fever, dry cough, and anorexia are similar to the presentation of acute sarcoidosis. In the setting of the current pandemic, this has caused delays in diagnosis [28]. CT of the chest with bronchoscopy and pathological sampling whenever necessary can obviate misdiagnosis and treatment, as was noted in the case of an emergency medical service pilot who was tested negative on the RT-PCR test and later showed non-caseating granulomas in the histopathology specimen from the sampled hilar lymph node [29].

### 4.1. Pathogenesis

The dysregulated immune response contributing to the pathophysiology of both COVID-19 and sarcoidosis raises certain concerns:

1. The defect in autophagy observed in patients with sarcoidosis might lead to the viral proteins bypassing the host defense mechanism causing an increased susceptibility to infection [30]
2. An aggravated immune response with Th1/Th17 activation at disease sites and peripheral lymphopenia is characteristic of sarcoidosis. Lymphopenia has also been observed in COVID-19 patients and has been considered a contributory factor to a worse prognosis [31]
3. The use of glucocorticoids and immunomodulators in sarcoidosis can further the immunosuppression leading to an increased susceptibility to infection
4. The odds of the SARS CoV-2 triggering a dysregulated antigen-host response in susceptible individuals leading to the initiation of granulomatous reaction and sarcoidosis is a possibility, with cases being reported of formation of sarcoid like granulomas in patients recovering from COVID-19 and the presence of multinucleate giant cells in resected lung specimens of patients infected with SARS-CoV2 [32,33]. The use of immunosuppressants, such as tocilizumab, has also been known to induce the unmasking of immune disorders such as sarcoidosis in susceptible individuals [34]
5. Viral infections have been contributory to the worsening of exacerbation in chronic respiratory diseases including sarcoidosis. The angiotensin-converting enzyme-2 (ACE-2), which counterbalances the effect of the ACE/renin angiotensin aldosterone pathway through the ACE-2/Ang1-7/MAS1 axis, is exclusively found in the lung epithelium and is known to be beneficial against lung fibrosis [35,36]. SARS-CoV2 induced downregulation of ACE-2 may promote worsening of interstitial fibrosis in patients with pulmonary sarcoidosis.

### 4.2. Radiology

Chest roentgenograms can detect the presence of mediastinal widening and reticulonodular pulmonary opacities in sarcoidosis, and based on the degree of involvement, has been classified by the Siltzbach classification system as stage 0, with a normal appearance; Stage 1, with lymphadenopathy only; Stage 2, with lymphadenopathy and parenchymal lung disease; Stage 3, with parenchymal lung disease only; and Stage 4, with pulmonary fibrosis [31]. However, chest radiography has only a limited utility in COVID-19. Bilateral consolidations or ground glass haze and reticular opacities may only be seen in advanced stages of COVID-19 [37,38].

The presence of mediastinal and/or bilateral hilar lymphadenopathy and peri-lymphatic nodular opacities with an upper lobe predilection on a CT is characteristic of pulmonary sarcoidosis [39]. While the presence of lower lobe predominant, peripheral patchy ground-glass opacities and interstitial thickening point toward COVID-19. The suspicion of lung involvement on CT scans in COVID-19 has been classified based on the COVID-19 Reporting and Data System (CO RADS) using a five-point scale ranging from 1 (very low suspicion) to 5 (very high) and 6 being proven infection microbiologically.

However, COVID-19 and sarcoidosis may rarely appear similar in radiology, and atypical features may occur in both, leading to a wrong diagnosis especially in asymptomatic cases [40]. Occasionally, in sarcoidosis, patients with pulmonary involvement may present with a dense perihilar consolidation and peripheral nodularity (10-20%), peripheral patchy ground-glass opacities (40%), cavitary (<0.8%), or miliary changes (<1%) [39]. Atypical features such as dense consolidation with cavitation, pleural effusion, and pericardial effusion may be seen in the intermediate phase of COVID-19 [41,42]. Features usually seen in pulmonary sarcoidosis such as reverse halo sign, patchy or nodular ground-glass opacities, consolidation, and crazy-paving pattern can be seen in COVID-19 as well. Rare cases have also been reported of hilar lymphadenopathy and nodular consolidations in COVID-19 in an elderly lady from COVID-19 infection and respiratory distress [43].

### 4.3. Clinical outcome

The rate of ICU admission reported in our analysis was 22.2%. A meta-analysis of ICU admission rates in COVID-19 showed a pooled ICU admission rate of 32% [44]. The lower values in our study may be attributed to lower sample size. In a registry from several French hospitals, of 36 patients, 78% were admitted to the hospital with a diagnosis of sarcoidosis and COVID-19 among which 36% required ICU admission and 14% died of complications [22]. Other studies have shown rates of ICU admissions to be between 3.4 and 7.8% and mortality rates between 1.3% and 16.2% (Table 2). Although the mere diagnosis of sarcoidosis was not associated with a significant risk of hospitalization or ICU admission when compared to others, as noted by Morgenthau *et al.*, the presence of moderate to severe abnormalities in lung function was a significant risk factor contributing to hospital admission, need for ICU care, and in-hospital mortality [23]. It was noted that among all patients with interstitial lung disease (ILD), the mean predicted functional vital capacity (FVC) and diffusing capacity of the lung for carbon monoxide (DLCO) for those who survived were 82.2% and 56.4%, respectively, while the mean predicted FVC and DLCO for those who died was 76.8% and 49.6%, respectively. In the largest multi-center retrospective follow-up so far of COVID-19 in pulmonary sarcoidosis by Hadi *et al.*, 954 patients with diagnosed COVID-19 were propensity score-matched with patients presenting with COVID-19 without sarcoidosis. No statistically significant difference was observed for mortality, inpatient admission, mechanical ventilation, critical care need, acute kidney injury or requirement for renal replacement therapy among the groups.

### 4.4. Management of sarcoidosis amidst the COVID-19 pandemic

Studies have demonstrated that ongoing glucocorticoids, methotrexate, and TNFa inhibitors do not pose a significant risk for acquiring a COVID-19 infection. However, the risk of infection was 5 times higher in patients receiving rituximab, as reported by Baughman *et al*. [26] It was also noted in his study that though hydroxychloroquine (HCQ) was once considered a prophylactic against SARS-CoV-2 infection, there was no significant change in the rates of infection or hospitalization in patients receiving HCQ. Among the 27 patients in our review, the odds of oxygen requirement, ICU admission, and death among patients who have been receiving steroids, immunomodulators, and TNF a inhibitors was found to be only 0.13%. Due to the low prevalence of sarcoidosis, there have been no large-scale comparative studies assessing the effect of steroids, immunomodulators, and biologicals on patients with sarcoidosis, in terms of severity of clinical presentation in COVID-19, differences in management, the incidence of secondary infection and outcome. Recommendations have been made only based on expert opinion and clinicians have been advised to exercise caution in a patient -to patient basis.

Steroids should not be abruptly stopped irrespective of exposure. However, in patients with stable sarcoidosis, the dose of steroids can be tapered up to 50% or the lowest possible dose. For patients receiving high doses of glucocorticoids >40 mg per day with organ threatening manifestations, the addition of disease-modifying drugs such as methotrexate may be considered [45]. Glucocorticoids, however, may pose a risk of delayed viral clearance, secondary infections, need for antibiotic use, and increase the possibility of relapse of COVID-19, as was seen in a 50-year-old man with Lofgren’s syndrome, who after the spontaneous remission of COVID-19, was initiated on prednisolone for ankle pain and swelling. He later tested positive again nearly a week after his previous negative test [11,46]. COVID-19 can also trigger a relapse of sarcoidosis in patients in remission. In acute pulmonary exacerbations of sarcoidosis, the previous treatment with corticosteroids is a significant risk factor [47]. Clinicians must, therefore, exert caution during initiation of steroids in newly diagnosed sarcoidosis and must do so only when indicated.

Disease-modifying drugs such as methotrexate, azathioprine, mycophenolate mofetil, and biologicals may be continued in patients with stable disease. However, reduction of the dose or frequency of administration may be attempted. In patients with clinically active or organ-threatening sarcoidosis, the same dose may be continued for the fear of life-threatening relapse [45]. Methotrexate may aggravate pneumonitis in patients admitted to the ICU. In our review, it has been observed that only one of the patients reported by Padala *et al*. had methotrexate and adalimumab being withheld during ICU admission, for the fear of further immune suppression, following which the patient had shown improvement in the next 6 days. In other studies, only the use of rituximab had shown increased rates of infection, but no difference in hospitalization rates was observed [7].

### 4.5. Management of COVID-19 in sarcoidosis patients

Because of the rapid spread of the COVID-19 global pandemic, there was little time to develop new therapeutic options. As a result, there was great interest in repurposing already existing medications for the treatment of COVID-19. One drug class that was explored in our review was tetracyclines, in particular, doxycycline. Yates *et al*. have described a case series of four patients with pre-existing lung disease who developed COVID-19 and responded to treatment with doxycycline [13]. One patient among the four was a 71-year-old white male with a 9-year history of sarcoidosis. He was treated with doxycycline for 10 days upon diagnosis of COVID-19, along with self-monitored pulse oximetry at home. Apart from a minor drop in oxygen saturation on day 6, the patient had an uneventful clinical course and was able to return to work after 3 weeks, when he tested negative for SARS-CoV-2. Supportive management, hospitalization, ICU care, non-invasive ventilation, or invasive mechanical ventilation may be inducted based on clinical indications. Non-invasive ventilation may help avert the need for intubation and its related complications especially in patients with mild ARDS [48]. Stepping up the dose of steroids may be done in appropriate situations such as sarcoidosis patients with known obstructive airway disease or septic shock [49]. Since ILD exacerbations and COVID-19 ARDS may share a similar radiological picture and histopathological feature of diffuse alveolar damage, it might pose a challenge to the treating clinicians [50]. Mechanical ventilation should be avoided in advanced lung involvement with fibrotic lung disease, where the mortality rate from acute exacerbation of ILD alone is around 50% and is likely to be worse with an additional diagnosis of COVID-19 [49].

Although sarcoidosis has the best 5-year survival rates among the ILD of 91.6% compared to 69.7% for connective tissue disease-related ILD and 35% for Idiopathic pulmonary fibrosis, one in every three patients may experience an acute exacerbation [51]. It has been noted from an ILD registry published during the pandemic that only 33% of patients admitted with sarcoidosis and ILD had died, which was second only to connective tissue ILD-excluding rheumatoid arthritis [52]. About 93% of the people with ILD who survived did not receive any advanced respiratory support in the form of intubation and mechanical ventilation, and 83% of those who did receive it, died.

To date, there is no systematic analysis of COVID-19 patients with sarcoidosis. One would expect a poor outcome for patients with sarcoidosis with COVID-19 for two reasons. Patients with sarcoidosis are likely to be on immunosuppressive medication. This may turn out to be a double-edged sword [45]. On the one hand, it may predispose to an increased risk of secondary infections. On the other, immunosuppressive medication may be beneficial as the lung injury in COVID-19 is mediated by the host immune responses [46]. Second, patients with pulmonary sarcoidosis are expected to have some degree of lung fibrosis, which would lead to faster deterioration and prolonged recovery if they develop COVID-19. There is also concern regarding COVID-19 being a trigger for acute exacerbation of sarcoidosis [11].

Studies conducted on the effect of immunosuppression in patients of sarcoidosis who develop COVID-19 have an element of bias in the form of hospital bias or lack of data regarding mortality in questionnaire-based surveys. Guidelines, so far, have been based on expert opinion and extrapolation of outcomes from studies involving patients with autoimmune diseases who receive similar medication. Large-scale studies, such as follow-up of sarcoidosis registries from multiple hospitals, need to be assessed to quantify the rates of infection, hospital admission, ICU requirement, mortality, and long-term sequelae among patients on active treatment and those in remission of sarcoidosis.

### 4.6. Limitation

Our review addresses the need to review our treatment protocols in newly diagnosed patients with sarcoidosis and stresses the need for further evidence regarding the possible risk versus benefit analysis of commonly used treatment protocols in sarcoidosis. Lack of data in some case reports regarding the staging of the disease, previous pulmonary function assessments or treatment regimens, and the overall small sample size may have led to unsettling conclusions and bias regarding outcomes. The low global prevalence of sarcoidosis may deter large-scale follow-up of patients, as it has been observed that most patients assessed in prospective studies are usually limited to a single center. Further studies are awaited in this regard. Studies also did not describe the interactions of comorbid conditions, medications, and adverse events in patients consistently. Most studies were limited in details of long-term outcomes as well.

## 5. Conclusion

The clinical presentation of sarcoidosis patients with COVID-19 is similar to the general population. Imaging and subsequent management also do not vary considerably when compared to non-sarcoidosis patients. Although, in patients with advanced lung fibrosis and poor quality of life, restraint from an aggressive approach in management may be advocated.

### Conflict of Interest

The authors declare no conflicts of interest.
